# In-depth mapping of human testicular and epididymal proteins and their functional association with spermatozoa

**DOI:** 10.3892/mmr.2015.3435

**Published:** 2015-03-05

**Authors:** XUEXIA LIU, FUJUN LIU

**Affiliations:** Central Laboratory, Yantai Yu Huang Ding Hospital, Qingdao University, Yantai, Shandong 264000, P.R. China

**Keywords:** testis, epididymis, sperm milieu, sperm maturation, immunohistochemistry

## Abstract

The mammalian testis and epididymis are responsible for spermatozoa production and maturation, which contributes to male fertility. Predominantly expressed proteins in the testis and epididymis were suggested to be involved in the key functions or pathways in spermatogenesis and sperm maturation. To further investigate these proteins and their associations with sperm, large protein profiles of human testis and epididymis were mapped. Predominantly-expressed testicular (173) and epididymal (244) secreted proteins were further screened and functionally characterized. Differential expression levels of solute carrier family 2 (facilitated glucose transporter), member 3, solute carrier family 25 (carnitine/acylcarnitine translocase), member 20, WAP-type four-disulfide core domain protein 8 and prostate and testis expressed 1 were validated using western blot and immunohistochemical analyses. The results may provide novel insight into the understanding of testicular and epididymal physiology and function, and facilitate sperm maturation research.

## Introduction

Mammalian spermatozoa are produced in the testis and mature in the epididymis; thus, the testis and epididymis determine male fertility. The testis generate non-functional sperm from precursor germ cells (1), which the epididymis subsequently matures, protects and stores prior to ejaculation (2,3). Testicular sperm is released into the seminiferous tubules, and progresses through the epididymal lumen. During this process, the sperm travels through highly specialized and localized microenvironments in the seminiferous tubules and epididymal lumen, which are maintained by the blood-testis and blood-epididymis barriers, respectively (4,5). The barriers provide protection for the sperm from toxic substances and the immune system (6). The microenvironment may directly contribute to spermatogenesis and epididymal sperm maturation, and consequentially affects the quality of the sperm (7). Thus, the secreted microenvironment proteins in the tubules are targets for interference, which may result in the disruption of spermatogenesis or sperm maturation, and provide the basis for the development of male contraceptive agents (8,9). Therefore, investigating the interactional association between the fluid microenvironment and sperm is clinically relevant.

Previous studies of adult human testicular and epididymal tissue and fluid (sperm-milieu) proteomes have characterized the testicular and epididymal proteins and their functions, in addition to suggesting the major pathways in which these proteins participate (10,11). Within the sperm-milieu proteome, 524 sperm-milieu proteins have been identified by two-dimensional gel electrophoresis and matrix-assisted laser desorption/ionization (MALDI)-time-of-flight (TOF) or MALDI-TOF/TOF mass spectrometry. Using the corresponding antibodies, 319 sperm-located domain-specific proteins of testicular and epididymal origin, were identified, which provided novel insight into the biology of spermatogenesis and sperm maturation. However, due to the limited data generated by current technologies, an overall insight still remains to be gained. The Human Protein Atlas (HPA; www.proteinatlas.org) has produced a global immunohistochemistry map of testicular and epididymal protein expression profiles (12), which provided a reliable resource for mapping testicular and epididymal protein profiles. The present study performed a direct comparison of protein expression levels in testicular and epididymal tissues to identify the predominantly expressed proteins in the testis and epididymis. In addition, a secretory protein profile was identified as a candidate for the creation of the sperm milieu, which bioinformatically appeared to be important in the maturation and survival of sperm. The present study aimed to conduct a detailed comparative mapping of testicular and epididymal protein profiles in order to extend current knowledge.

## Materials and methods

### Sample preparation

The testes and epididymides were obtained from five young fathers (27–33 years old), who had died in car accidents with no history of pathology that may have affected their reproductive function, and had consented to donate their bodies to medical research whilst they were alive. The donation of their organs for medical research was additionally approved by their immediate family. All procedures were approved by the Ethics Committee of Yu Huang Ding Hospital (Yantai, China). The organs from one side were processed for protein extraction and the other for immunohistochemistry. Protein extraction was conducted as previously described (10,11).

### Data collection

Staining profiles for proteins in the human testis and epididymis were downloaded from the Human Protein Atlas (http://www.proteinatlas.org). The expression levels of each protein were graded into four levels: Strong, moderate, weak and negative. The differentially-expressed proteins referred to proteins with a change of >2 classification levels between the testis and epididymis, and the resulting proteins were referred to as predominantly-expressed testicular or epididymal proteins. A current complet data set of human seminal fluid proteins was built by integrating the published proteomic works of seminal fluids (13). These proteins, combining analysis with the secretory prediction tool Locate (http://locate.imb.uq.edu.au/), were used as expected secreted proteins to screen the epididymal fluid proteins. A list of human sperm proteins was obtained by collecting data from a previous study (14).

### Gene ontology (GO) analysis

The general functions of predominantly expressed testicular, epididymal and sperm milieu proteins were broadly classified according to the GO annotation (www.geneontology.org) and protein class annotation in Panther (http://www.pantherdb.org).

### Overrepresentation analysis of predominantly-expressed proteins

Overrepresentation analysis of the GO terms, including biological processes and molecular functions, was conducted using ConsensusPathDB-human (http://cpdb.molgen.mpg.de/CPDB), which is a molecular functional interaction database. GO level 2 and 3 categories were selected, and the P-value cutoff was set as 0.01.

### Validation experiment

Western blot and immunohistochemical analyses were performed as previously described (10,11). The primary antibodies were as follows: Goat polyclonal anti-solute carrier family 2 (facilitated glucose transporter), member 3 (SLC2A3) (sc-31838; Santa Cruz Biotechnology, Inc., Dallas, TX, USA), goat polyclonal anti-solute carrier family 25 (carnitine/acylcarnitine translocase), member 20 (SLC25A20) (sc-103220; Santa Cruz Biotechnology, Inc.), rabbit polyclonal anti-WAP-type four-disulfide core domain protein 8 (WFDC8) (sc-86261; Santa Cruz Biotechnology, Inc.) and rabbit polyclonal anti-prostate and testis expressed 1 (PATE1) (ab173522; Abcam, Cambridge, MA, USA). Briefly, 50 *μ*g protein was separated by 12.5% (w/v) SDS-PAGE and was then transferred to polyvinylidene difluoride membranes (Sigma-Aldrich, St. Louis, MO, USA) for western blotting. The membranes were blocked with 3% (w/v) non-fat milk (Wondersun Dairy Co., Ltd., Harbin, China) for 1 h and were incubated with the respective primary antibody at room temperature for 1 h. Following washing with Tris-buffered saline and Tween-20^®^ (Sigma-Aldrich) three times, membranes were incubated with horseradish peroxidase (HRP)-conjugated anti-immunoglobulin G (OriGene Technologies, Inc., Beijing, China) for 1 h. A diaminobenzidene (DAB) kit (OriGene Technologies, Inc.) was used to visualize the immunoreactive complexes and the membranes were scanned with a Z320 scanner (Founder, Beijing, China). For immunohistochemistry, the tissues were fixed in Bouin’s solution (Sigma-Aldrich) for 10 h and embedded with paraffin. Sections (4 *μ*m) were incubated in a microwave oven for 15 min for antigen retrieval. Incubation of the sections with H_2_O_2_ (3%; v/v) (Science & Technology Co., Ltd, Yantai, China) for 10 min was used to to remove endogenous peroxidases. Following antigen blocking with 3% bovine serum albumin (Sigma-Aldrich) for 30 min, the appropriate primary antibody was added to the sections overnight at 4°C. Following washing of the sections with Tris-buffered saline several times, HRP-conjugated anti-rabbit IgG (OriGene Technologies, Inc.) was added for 1 h at 37°C. The DAB kit was used to reveal the binding sites, and subsequently the sections were counterstained by hematoxylin (Abcam) and mounted for bright-field microscopy (DM LB2; Leica Microsystems GmbH, Nussloch, Germany).

### Statistical Analysis

Data are presented as the mean ± standard deviation. Means were compared between two groups using Student’s t-test. A commercial software package (SPSS 18.0; SPSS, Inc., Chicago, IL, USA) was used to perform the analysis. P<0.05 was considered to indicate a statistically significant difference.

## Results

### Protein profiles of human testis and epididymis

Proteins with annotations were retrieved from the Human Protein Atlas database (http://proteinatlas.org). The resulting data included 7,595 proteins expressed in the testicular seminiferous duct cells and 7,573 proteins in epididymal glandular cells. A total of 5,190 sperm protexins were identified from previous studies; of these, 1,309 and 1,308 proteins were observed to overlap in the testicular or epididymal samples, respectively.

### Differentially expressed testicular and epididymal proteins

Secretory proteins: The integrated seminal fluid proteins and secreted proteins identified by the prediction tool were retrieved to select potential target secretory proteins in human testis and epididymal expressed proteins. A total of 2,960 proteins were obtained as the background secretory protein dataset. A total of 1,306 and 1,377 potential secretory proteins were identified in the testis and epididymis, respectively.

Human testicular and epididymal predominant expression proteins: Proteins in the HPA were graded into four expression level categories: High, moderate, low and negative. A total of 2,451 proteins exhibited high expression levels in the testis and 2,218 proteins exhibited high expression levels in the epididymis. By comparing the protein expression levels between testicular and epididymal samples, a total of 2,783 proteins were demonstrated to have differential expression levels. By further screening with a strict criterion of a two-fold difference in expression, 1,412 proteins were identified to be predominantly expressed in the testis, including 388 sperm proteins and 173 secretory proteins. A total of 1,371 proteins were observed to be predominantly expressed in the epididymis, including 363 sperm proteins and 244 secretory proteins (Table I). These secreted proteins predominantly expressed in human testis or epididymis were suggested as promising sperm milieu proteins as previously described (10,11). Of the 244 secretory proteins identified in the epididymis, 56 were common with the sperm milieu identified in a previous study.

### Functional analysis

**Figure 1:**
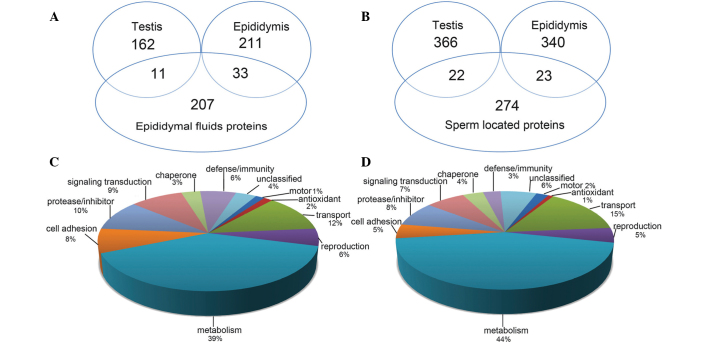
Distribution and functional analysis of predominantly expressed secreted and sperm-located proteins. Distribution analysis of (A) secreted and (B) sperm-located proteins. Functional analysis of (C) secreted and (D) sperm-located proteins.

Broad ontological analysis was performed on the secreted and sperm-located proteins that were predominantly expressed in the testis and epididymis. All the proteins were placed into several broad functional categories on the basis of the GO and Panther databases. As demonstrated in Fig. 1, the majority of proteins were involved in metabolic functions, followed by protease/inhibitor (10%), cell adhesion (8%), defense/immunity (6%) and antioxidant (2%) functions in the secreted proteins, as well as transport (15%) and motor (2%) functions in the sperm-located proteins.

Further overrepresentation analysis was conducted on the predominantly-expressed proteins in the testis, epididymis and epididymal fluids. The enriched terms reflected their main functions in spermatogenesis and sperm maturation. Predominantly-expressed testicular proteins were observed to be mainly involved in the functions of germ cell development and differentiation, whereas proteins in the epididymis functioned predominantly in cell adhesion associated with epithelial cells. Epididymal fluid proteins were identified to have significant enzymatic activity (Table II).

### Validation of protein expression in the human testis and epididymis by western blotting and immunohistochemistry

**Figure 2:**
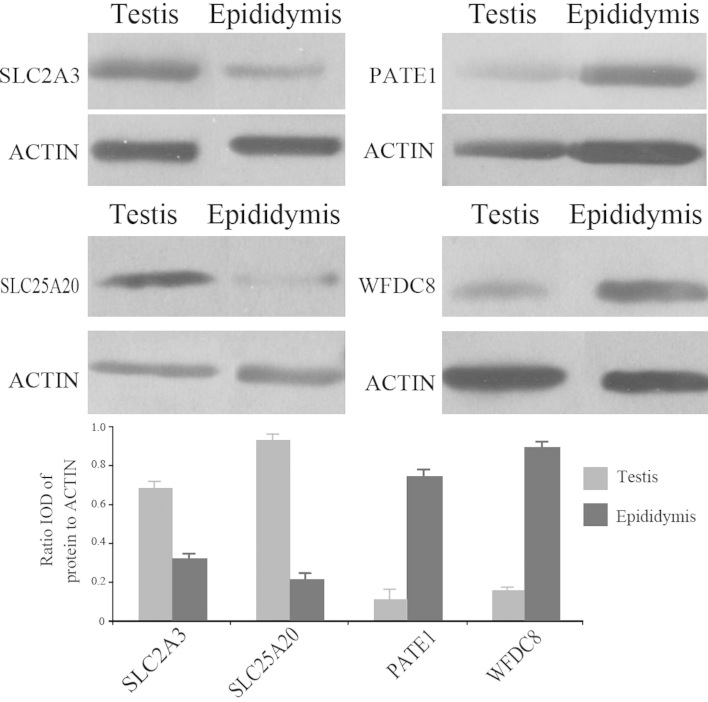
Western blot analyses of expression levels of SLC2A3, SLC25A20, PATE1 and EFDC8 in human testis and epididymis. IOD, integrated optical density; SLC2A3, solute carrier family 2 (facilitated glucose transporter), member 3; SCL25A20, solute carrier family 25 (carnitine/acylcarnitine translocase), member 20; WFDC8, WAP-type four-disulfide core domain protein 8; PATE1, prostate and testis expressed 1.

**Figure 3:**
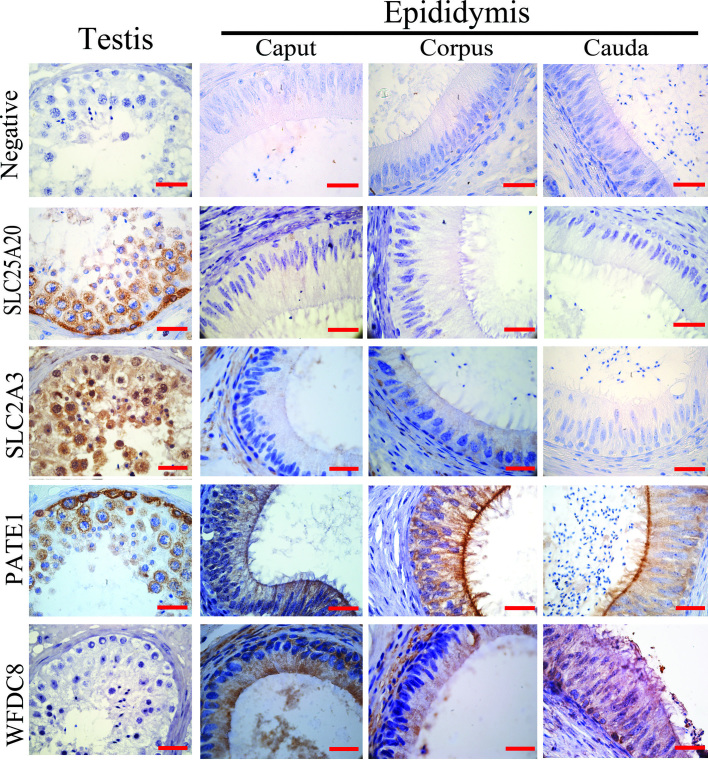
Immunohistochemical localization of SLC2A3, SLC25A20, PATE1 and WFDC8 in the human testis and epididymis. Scale bar, 40 *μ*m. SLC2A3, solute carrier family 2 (facilitated glucose transporter), member 3; SCL25A20, solute carrier family 25 (carnitine/acylcarnitine translocase), member 20; WFDC8, WAP-type four-disulfide core domain protein 8; PATE1, prostate and testis expressed 1.

Four proteins (SLC2A3, SLC25A20, WFDC8 and PATE1), involved in the functions of transport and signal transduction, were randomly selected for validation by western blotting. The results indicated higher expression levels of SLC2A3 and SLC25A20 in normal human testis, and higher expression of WFDC8 and PATE1 in the human epididymis (Fig. 2). In agreement with the results from the western blot analysis, SLC2A3 and SLC25A20 were observed to exhibit strong staining intensity in the testis with positive staining in the spermatogonium and spermatocyte, whereas WFDC8 and PATE1 were strongly expressed in the human epididymis, particularly in the principle cells of corpus epididymis (Fig. 3).

## Discussion

Mammalian testis and epididymis have blood-testis and blood-epididymis barriers, which create a distinct microenvironment for spermatogenesis and sperm maturation (15). The fluid environments in the seminiferous tubules are predominantly produced by the seminiferous cells, while those in the epididymal lumen are mainly created by principle cells (4,16). During the progression of sperm along the testicular and epididymal tubules, they interact with fluid factors, particularly proteins, in order to complete their modifications (17). A previous study mapped the human testicular and epididymal protein profile and investigated their associations with the sperm proteome by traditional two-dimensional separation coupled with identification by MALDI-TOF (10,11). However, the number of identified proteins by these studies was limited. In order to further elucidate these interactional associations, data were retrieved from the HPA in the present study to compare protein expression levels in the testis and epididymis, and thereby, the identity of several secretory proteins and their associations with sperm were determined.

In the present study, a total of 7,595 and 7,573 proteins with immunohistochemistry values in the testis and epididymis tissues, respectively, were retrieved from the HPA database. These proteins were used to perform comparative analysis between the testis and epididymis. The amount of data collected was markedly greater than the proteomic data currently available (10,18,19), and may thus provide an improved understanding of testicular and epididymal protein profiles. Subsequently, predominantly-expressed proteins in the testis or epididymis were screened by comparing differential expression levels between two datasets, and the results were consistent with the reported transcriptomic elevation (20). It was hypothesized that the predominantly expressed proteins in the testis would serve important roles in spermatogenesis, and those in the epididymis would have important functions in the maturation of sperm. Enrichment functional analysis demonstrated that predominantly expressed proteins in the testis were mainly involved in the functions of germ cell development and differentiation, and those in the epididymis mainly functioned in cell adhesion associated with epithelial cells. The enrichment functions were consistent with the roles of the corresponding tissues. Thus, in future studies, further data mining analysis may be performed based on these data.

Of these proteins, highly expressed secretory proteins, particularly epididymal secretory proteins, were investigated due to their direct association with spermatogenesis and sperm maturation. In the present study, secretory proteins were further identified by combined retrieval of seminal fluid proteins and a secretory prediction tool (Locate). These proteins were suggested to be secreted into the testicular tubules or the epididymal lumen, thus contributing to the microenvironment (sperm milieu). Data from the present study suggested that secretory proteins highly expressed in the testis and epididymis were predominantly involved in the creation of the testicular and epidymal microenvironments, respectively. With the exception of metabolic activity, the secreted proteins were observed to be predominantly involved in protease/inhibitor, cell adhesion, defense/immunity and antioxidant functions, which is consistent with the results of a previous study (21). The proteases and inhibitors were differentially expressed in testicular and epididymal fluids, suggesting different roles in spermatogenesis and sperm maturation (22). Defense/immunity and antioxidant proteins may serve cooperative functions in sperm survival (23). Epididymal fluids were directly involved in sperm maturation, and 251 human epididymal fluid proteins were identified by 2D-gel separation combing identification by MALDI-TOF mass spectrometry (11). Secreted proteins in the present study were identified as potential novel members of the epididymal sperm milieu. These proteins were involved in various functions which contribute to a continual and appropriate microenvironment for sperm maturation, transit and storage.

In conclusion, the present study provided novel insight into the human testis and epididymis proteomes, and bioinformatically characterized the predominantly expressed tissue proteins and secreted proteins. The candidate secreted proteins and enriched functions require further investigation, which will aid in a more thorough understanding of the male reproductive proteome, and additionally facilitate research on sperm maturation.

## Figures and Tables

**Table I:** Characteristics of testicular and epididymal expressed proteins.

Expression	Weak (n)		Moderate (n)		Strong (n)		Predominant (n)		
	Sperm	Secretory	Sperm	Secretory	Sperm	Secretory	Sperm	Secretory	Others
Testicular	0	121	849	745	460	440	388	173	969
Epididymal	0	118	871	815	437	444	363	244	928

Data were retrieved from Human Protein Atlas; 2451 testis proteins and 2218 epididymis proteins were categorized, respectively. n, number of proteins.

**Table II:** Overrepresentation analysis of proteins predominantly expressed in the testis and epididymis.

A, Predominantly expressed testicular proteins		
GO term	Candidates contained, n (%)	P-value
GO:0007276 gamete generation	84 (15.8)	1.89×10^−12^
GO:0007126 meiosis	29 (17.7)	4.04×10^−06^
GO:0048515 spermatid differentiation	19 (22.4)	5.83×10^−06^
GO:0051321 meiotic cell cycle	29 (17.3)	6.60×10^−06^
GO:0009566 fertilization	21 (19.3)	2.22×10^−05^
GO:0007281 germ cell development	24 (17.1)	4.50×10^−05^
GO:0030317 sperm motility	9 (27.3)	3.53×10^−04^
GO:0003188 heart valve formation	5 (45.5)	5.67×10^−04^
GO:0007049 cell cycle	130 (9.2)	1.14×10^−03^
GO:0007548 gender differentiation	31 (12.3)	1.98×10^−03^
GO:0007128 meiotic prophase I	6 (28.6)	2.67×10^−03^
GO:0051301 cell division	51 (10.5)	3.33×10^−03^
GO:0007530 gender determination	6 (25.0)	5.53×10^−03^
GO:0048608 reproductive structure development	30 (11.3)	7.33×10^−03^
GO:0035036 sperm-egg recognition	5 (26.3)	8.87×10^−03^

B, Predominantly expressed epididymal proteins		
GO term	Candidates contained, n (%)	P-value

GO:0007155 cell adhesion	91 (9.4)	3.37×10^−03^
GO:0042221 response to chemical stimulus	241 (8.2)	5.74×10^−03^
GO:0030855 epithelial cell differentiation	34 (11.1)	5.88×10^−03^
GO:0006612 protein targeting to membrane	20 (12.8)	6.95×10^−03^
GO:0051641 cellular localization	178 (8.4)	7.49×10^−03^
GO:0008104 protein localization	142 (8.6)	7.50×10^−03^

C, Epididymal fluid proteins		
GO term	Candidates contained, n (%)	P-value

GO:0006629 lipid metabolic process	34 (2.9)	1.64×10^−05^
GO:0009056 catabolic process	47 (2.3)	1.09×10^−04^
GO:2000145 regulation of cell motility	16 (3.7)	1.89×10^−04^
GO:0008233 peptidase activity	19 (3.2)	3.37×10^−04^
GO:0004857 enzyme inhibitor activity	13 (4.0)	4.03×10^−04^
GO:0006979 response to oxidative stress	10 (3.9)	2.10×10^−03^
GO:0006508 proteolysis	24 (2.4)	3.02×10^−03^
GO:0009566 fertilization	6 (5.5)	3.21×10^−03^
GO:0051604 protein maturation	7 (4.2)	6.51×10^−03^
GO:0050776 regulation of immune response	16 (2.5)	9.20×10^−03^

GO, gene ontology.
